# Reviewing the Dissociative Symptoms in Patients With Schizophreniaand their Association With Positive and Negative Symptoms

**Published:** 2014

**Authors:** Abolfazl Ghoreishi, Zahra Shajari

**Affiliations:** 1 Associate Professor, Department of Psychiatry, School of Medicine, Zanjan University of Medical Sciences, Zanjan, Iran.; 2 Scientific Researcher, Metabolic Disease Research Center, Zanjan University of Medical Sciences, Zanjan, Iran.

**Keywords:** Dissociative Experience Scale, Dissociative Symptom, Positive and Negative Syndrome Scale, Schizophrenia

## Abstract

**Objective:** The present study aimed to clarify dissociative symptoms in patients with schizophrenia and its association with negative and positive symptom of schizophrenia.

**Methods:** Based on the 4^th^ edition of Diagnostic and Statistical Manual of Mental Disorders (DSM-IV) criteria, 80 schizophrenic subjects were randomly selected from patients who referred to the clinics of psychiatry hospital in Kerman, Iran. Eighty non-schizophrenic patients were chosen as the control group. Both groups were evaluated for dissociation symptom using the Dissociative Experience Scale (DES). Positive and Negative Syndrome Scale (PANSS) score was also used in the case group for determination of positive and negative symptom of schizophrenia.

**Results: **The prevalence of dissociation symptom was 13% and 4% among schizophrenic and control groups, respectively (p = 0.02). In addition, there was a statistical significant association between DES score and positive symptom in schizophrenia (p = 0.02).

**Conclusion:** The association between dissociative symptom and schizophrenia was significant and dissociative symptoms were associated with positive symptoms of schizophrenia.

**Declaration of interest:** None.

## Introduction

Schizophrenia is a clinical syndrome with involvement of thought, perception, emotion, movement and behavior. Its prevalence has been reported from 1 to 1.5%. The ratio of suffering is the same for both males and females. The cause of this disease is obscure. However, there are some assumptions regarding its etiology such as genetic factors, neuro-immuno-virology, and complications during pregnancy and delivery (1).

Crow classified the schizophrenic patients into two types of I and II on the basis of inclusive or exclusive of positive or negative symptom. However, this system has not been approved as a part of the 4^th^ edition of Diagnostic and Statistical Manual of Mental Disorders (DSM-IV) (2-4).

The classification of schizophrenia into two types has considerably influenced the psychiatric research. Positive symptoms included hallucination and delusion, while the negative symptoms included superficial emotion, difficulty in finding words, obstruction in speech, lack of adornment, lack of motivation, and lack of feeling of pleasure or satisfaction and unsociability. Positive and Negative Syndrome Scale (PANSS) test is a standard method in examining schizophrenic patients (2, 5). Plus positive or negative symptoms, hysterical symptoms are also common in schizophrenic breakdown. Therefore, the presence of hysterical-dissociative, even convertible symptoms cannot reject the diagnosis of schizophrenia (5).

The term "dissociation" refers to a disorder in normal functioning of perception, memory, identity or consciousness (6). Although, dissociation is a distinguished symptom of dissociative disorders in the DSM, patients with other mental disturbances develop dissociative symptom as well. For example, the symptomatology of dissociation in samples of patients with schizophrenia and schizoaffective disorder has been reported over 50% (7).

The study of dissociation in schizophrenic patients is mainly due to high traumatic confrontation in patients with severe mental breakdown (8). It was found out that 98% of severe mental patients have been once confronted with traumatic incident during their lifetime or abused in their childhood leading to severe positive type of schizophrenia in adulthood (9). Moreover, some reports indicated the existence of high level of dissociation symptom in schizophrenic patients that was in association with other psychopathological disorders (10).

Although the association between trauma and dissociation has been well-known in patients with dissociative disorders and post trauma stress disorders, and even though patients with severe mental breakdown have higher traumatic confrontations, there are a few studies available regarding the association between trauma and dissociation in those patients.

Some studies indicated that there is an association between people’s personal reports of neglect or abuse in childhood and dissociative symptom in adulthood schizophrenia. Emotional abuse has high positive correlation with observed dissociation in schizophrenic population. Such findings describe some genetic predispositions to schizophrenia that may be in connection with disorder of correspondence mechanism. Such process exposes children with pre-schizophrenia to higher impacts of stress (11). Thus, children, who are at risk of schizophrenia, may be more liable to suffer from dissociation as well. Sometimes, the critical cases of dissociation may be misdiagnosed as schizophrenia. In fact, it is not uncommon for patients with posttraumatic stress disorder or dissociative identity disorder to receive a misdiagnosis of schizophrenia (12). Only upon the discovery of a precipitating event are such patients reassessed and assigned proper diagnoses and treatments (13). Nevertheless, investigation of premorbid trauma and ruling out of posttraumatic stress disorder (PTSD) is not a routine occurrence in the course of psychiatric diagnosis (14).

The only way for discovering facilitator events in such patients is re-examination, and checking diagnosis and prior to treatments. Thus, identification of dissociative symptoms in patient with schizophrenia and their association with the negative and positive symptoms of schizophrenia is important.

## Materials and Methods

***Study subjects***

In this descriptive study, the patients were chosen in a nine-month period in 2002. Two groups of subjects (schizophrenic patients and normal volunteers) were recruited from Shahid Beheshti Hospital, Kerman, Iran. Eighty schizophrenic patients aged 18-74 years were included, whose diagnosis was confirmed by clinical interview according to the DSM-IV criteria. Exclusive criteria were serious psychotic disorders other than schizophrenia, mental retardation, substance and/or alcohol abuse, and antiepileptic treatment. Eighty volunteers were selected from workers and employees of hospital as control group who had neither schizophrenic nor other major physiological or physical disorders (matched for gender and age). They were reassessed by a psychiatrist to confirm their physiological status. 

After explanation the study objectives and obtaining informed consent, the participants were re-interviewed on the basis of the DSM-IV scale.

***Instruments***

The patients were assessed by the PANSS (Positive and Negative Syndrome Scale) for their positive and negative symptoms by a trained psychiatrist. PANSS is a 30-item scale with 1 to 7 points (normal to extremely abnormal) for each item and sub-scores for 7 positive (P), 7 negative (N), and 16 global psychopathological symptoms i.e. delusions, formal thought disorders, hallucinations, agitation, grandiosity, suspiciousness/persecution, and hostility(P1 to P7); blunted affect, emotional withdrawal, poor rapport, social passivity and apathy, difficulty in abstract thinking, lack of spontaneity and flow of conversation, and stereotyped thinking (N1 to N7); and general (G) items such as health concerns, anxiety, guilt, tension, mannerisms and posturing, depression, motor retardation, uncooperative behavior, unusual thought contents, disorientation, poor attention, lack of judgment and insight, a volition, poor impulse control, self-centeredness, and active social avoidance (G1 to G16). (15). PANSS questionnaire was used in original version in English by a trained psychiatrist (i.e., the first author).

In PANSS theory, and in order to estimate positive and negative symptoms in schizophrenia, a classification was introduced by Crow TJ (2, 3). According to this classification, symptoms of schizophrenic patients are defined as three types as below: 

Type I is positive-dominant and defined as three or more positive symptoms and less than three negative symptom. Type II is negative-dominant, and type III is the mixed type. 

Then, the patients were evaluated through the DES (Dissociative Experience Scale). DES II questionnaire has been used since 1993 after being introduced by Putnam and Carlson as a highly valid and reliable test for screening dissociative experiences with 28 questions covering 4 subscales, which reflects constructs of dissociation symptoms: amnesia, depersonalization/derealization, absorption and imaginative involvement and passive influence. The test was translated to the Persian by the authors and proved by three psychiatrists for its fluency in Persian and English. Its content validity was also approved by the same psychiatrists. Reliability of the questionnaire was examined by split test and Cronbach’s alpha (split-half reliability coefficient for alternate questions = 0.93; split-half reliability coefficient for the first and second half of questions = 0.79; and Cronbach’s alpha coefficient = 0.95) (16-19). 

The collected data were analyzed using the SPSS for Windows 11.5 (SPSS Inc., Chicago, IL, USA) and Software Minitab. Such statistical procedures as craft-tab tables, spearman coefficient of correlation, ANOVA (analysis of variance) and regression were used accordingly.

## Results

Two groups of schizophrenic and control were matched for age and sex. In schizophrenic group, mean age of men and women were 37.6 ± 31.2 and 33.9 ± 8.68 years old, respectively. Most of the subjects (41.9%) were between 20 and 30 years old and 68 % of them were males. Mean age of the control group was 36 ± 10.9 and 34.1 ± 6.8 among 41% males and 59% females, respectively.

In general, the results showed that DES score in 8.1% was above 30, while score in 147 (91.9%) was below, and 30.58 cases (%36.3) received scores below 10 and 89 (%55.6) received scores between 10 and 30. 

**Figure1:**
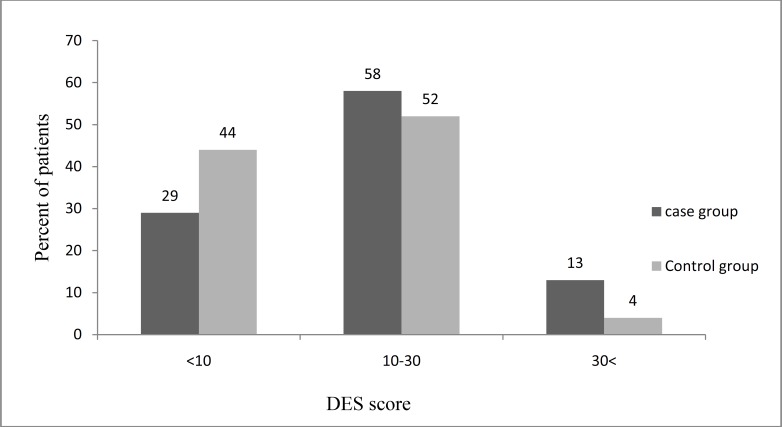
Distribution of dissociative experience scale (DES) score among schizophrenic patients and normal groups

Data analysis revealed that DES score in 23 (29%), 47 (58%), and 10 (13%) of the schizophrenic subjects was below 10, between 10 and 30, and above 30, respectively, whereas in the control group, it was 35 (44%), 42 (52%), and 3 (4%), respectively. This result showed that there was a statistical significant association between schizophrenia and dissociation (p = 0.03) (Figure 1).

Distributions of positive, negative, and mixed symptoms according to the gender of the schizophrenic patients are illustrated in table 1. There was a statistical significant association between DES scores and symptoms of schizophrenia. The association between the positive symptoms in schizophrenia (subdivisions of PANSS test) and DES score was statistically significant (p = 0.01), while no significant association was found between the negative and dissociative symptom.

Our results demonstrated that DES score was not changed significantly within two genders (0.47; p = 0.18) or age groups comparison (0.26; p = 0.32). 

Furthermore, an analysis of the subdivision of PANSS showed a statistical significant association of supplemental, general psychopathology, depression and activation with DES score.

**Table1:** Distribution of positive and negative symptoms based on Crow’s criteria in 80 schizophrenic patients

**Gender**	**Positive symptoms**		**Negative symptoms**		**Mixed symptoms**		**Total**
	**n**	**%**	**n**	**%**	**n**	**%**	**-**
**Male**	19	23.75	35	43.75	15	18.75	69
**Female**	02	02.50	06	07.50	03	03.75	11
**Total**	21	26.25	41	51.25	18	22.50	80

## Discussion

Our results indicated that there was a statistical significant association between dissociative symptom and schizophrenia. The prevalence of dissociative symptom in schizophrenic patients, on the basis of DES test criteria, was significantly higher than the control group. Findings of this research are accordance with those of Spitzer et al. (10) and some other studies (9, 12) which confirmed significant accompaniment of schizophrenia with dissociative symptoms. In contrary, Brunner et al. showed that there was no difference in the degree of reported dissociative experiences between schizophrenic patients and normal volunteers and patients with schizophrenia did not exhibit higher levels of dissociative symptoms in comparison to controls (18).

There is empirical evidence, primarily from the DES studies, that schizophrenic patients meet dissociative phenomena more often and to a higher degree than normal individuals, but with lower intensity and frequency than patients with borderline personality disorder (BPD), post-traumatic stress disorder (PTSD) and dissociative identity disorder(DID) (20).

The association between dissociation and psychosis may also be somewhat more important than recently thought. Dissociative symptom maybe is similar to psychotic symptom in many instances. More severe forms of dissociation may even be misdiagnosed as schizophrenia. In fact, it is not uncommon for patients with PTSD or DID to receive a misdiagnosis of schizophrenia (14, 21). Only upon the discovery of a precipitating event are such patients reassessed and assigned proper diagnoses and treatments. Nevertheless, investigation of pre-morbid trauma and ruling out of PTSD is not a routine occurrence in the course of psychiatric diagnosis (14, 21). Based on these findings, we suggest that a thorough screening for history of traumatic experiences should be incorporated into diagnostic procedures, and the possibility of alternate or co-morbid diagnoses entertained. Given the high proportion of severely mentally ill patients who have been exposed to traumatic events in childhood, Holowka et al. suggested that greater attention might be paid to dissociative tendencies in schizophrenia patients (13). 

The association between the emotional trauma and dissociation has been shown in several studies. In majority of these studies, dissociation was significantly associated with emotional abuse and physical abuse in childhood (13, 22, 23). The major finding of the present studies was that of the five distinct forms of maltreatment reported, emotional abuse during childhood was most strongly associated with the dissociative symptoms presented in adult schizophrenia patients (13, 24).

We found a statistically significant association between positive symptoms and dissociative experience; whereas there were not any association between negative and dissociative symptoms and dissociative experience. Similar to our results which indicated significant association between DES scores and positive and negative symptoms in schizophrenia, Spitzer et al. (10) found a close association between dissociative symptoms and positive symptoms as measured by the PANSS. They showed that patients with positive symptoms had significantly higher DES mean scores as compared to the patients with a predominance of negative symptoms. 

## Conclusion

The results of the present study provided supportive evidence that dissociative symptoms are also strongly associated with a schizophrenic disorder without gender or age preference and this significant association was observed with positive symptoms in schizophrenia.

Therefore, future investigations are required to define the etiologies of dissociation and the exact time of assessment of dissociative symptoms.
